# Environmental factors associated With *Toxoplasma gondii* Exposure in Neotropical Primates of Costa Rica

**DOI:** 10.3389/fvets.2020.583032

**Published:** 2020-10-22

**Authors:** Carmen Niehaus, Manuel Spínola, Chunlei Su, Norman Rojas, Oscar Rico-Chávez, Carlos N. Ibarra-Cerdeña, Janet Foley, Gerardo Suzán, Gustavo A. Gutiérrez-Espeleta, Andrea Chaves

**Affiliations:** ^1^Escuela de Biología, Universidad de Costa Rica, San Pedro, Costa Rica; ^2^Posgrado Regional en Ciencias Veterinarias Tropicales, Universidad Nacional de Costa Rica, San Pedro, Costa Rica; ^3^Instituto Internacional de Conservación y Manejo de Vida Silvestre, Universidad Nacional de Costa Rica, San Pedro, Costa Rica; ^4^Department of Microbiology, College of Arts and Sciences, University of Tennessee, Knoxville, Knoxville, TN, United States; ^5^Centro de Investigación en Enfermedades Tropicales, Facultad de Microbiología, Universidad de Costa Rica, San Pedro, Costa Rica; ^6^Departamento de Etología, Fauna Silvestre y Animales de Laboratorio, Facultad de Medicina Veterinaria y Zootecnia, Universidad Nacional Autónoma de México, Mexico City, Mexico; ^7^Departamento de Ecología Humana, Centro de Investigaciones y de Estudios de Avanzados del IPN (Cinvestav), Unidad Mérida, Mérida, Mexico; ^8^Department of Medicine and Epidemiology, School of Veterinary Medicine, University of California, Davis, Davis, CA, United States

**Keywords:** serology, *Alouatta palliata*, *Ateles geoffroyi*, *Cebus imitator*, *Saimiri oerstedii*, endoparasites, Latin America

## Abstract

The apicomplexan parasite *Toxoplasma gondii* (*T. gondii*) has been found in more than 350 species of homoeothermic vertebrates in diverse climates and geographic areas. In most animals, *T. gondii* produces mild or asymptomatic infection. However, acute and hyperacute toxoplasmosis is associated with high mortality rates observed in Neotropical primates (NP) in captivity. These primates are distributed in 20 countries across the Americas, and although infection has been reported in certain countries and species, toxoplasmosis in the wild and its impact on NP population survival is unknown. Differences among species in exposure rates and disease susceptibility may be due in part to differences in host behavior and ecology. Four species of NP are found in Costa Rica, i.e., howler (*Alouatta palliata*), spider (*Ateles geoffroyi*), capuchin (*Cebus imitator*), and squirrel monkeys (*Saimiri oerstedii*). This study reports NP exposure to *T. gondii* using the modified agglutination test in 245 serum samples of NP (198 wild and 47 from captivity) from Costa Rica. Associations of serostatus with environmental (forest cover, annual mean temperature), anthropogenic (human population density), and biological (sex) variables in howler and capuchin monkeys were evaluated. The seroprevalence among wild NP was 11.6% (95% CI = 7.7–17.34), compared with 60% in captive monkeys (95% CI = 44.27–73.63), with significant differences between species (*X*^2^ = 20.072; df = 3, *p* = 0.000164), suggesting an effect of behavior and ecology. In general, antibody titers were low for wild NP (<1:128) and high for captive NP (>1:8192), suggesting higher exposure due to management factors and increased life span in captivity. Seropositivity in howler monkeys was positively related to forest cover and inversely related to annual rainfall. For capuchins, annual rainfall was inversely related to seropositivity. Surveillance of *T. gondii* exposure in NP in captivity and in the wild is required to understand drivers of the infection and develop novel strategies to protect them.

## Introduction

*Toxoplasma gondii* (*T. gondii*) is an obligate intracellular protozoan parasite that can infect more than 350 species of mammals and birds worldwide (1–3). It is the single species within its genus, in the phylum Apicomplexa (4). The parasite (5) undergoes sexual reproduction to produce oocysts in most domestic and wild felids, which function as definitive hosts and excrete oocysts in their feces. Oocysts are the infective stage for intermediate hosts, and thus, felid latrines are a source of contamination for intermediate hosts (6). *T. gondii* can also be transmitted via ingestion of encysted organisms in tissue (7), allowing the parasite to bio-accumulate in intermediate hosts such as carnivores and scavengers. In addition, several species of cockroaches, earthworms, and beetles function as mechanical hosts (1, 8).

Seroprevalence of *T. gondii* antibodies in hosts varies spatially (9) and temporally, and is influenced by climate (9, 10). For example, higher seroprevalence has been observed during years with high temperatures or high rainfall in humans (11), rabbits (12), wild ruminants (13), and domestic cats (9, 14, 15). Infection risk is often moderated through impacts on the distribution of domestic and wild cats, which are influenced by human factors such as the presence of domestic and peridomestic rodents, intra or inter specific territorial interactions, environmental stress, vegetation, and landscape characteristics (16).

Host-specific factors also influence susceptibility to *T. gondii*, with higher prevalence sometimes detected in larger rodents and lagomorphs than smaller ones (17), and in species with a longer life expectancy (18). This may be due to greater exposure to oocysts in species with larger home ranges, longer life expectancies, and higher energy requirements, which are related to body size (19). Males with larger body masses may also consume more and larger prey than females (20), reflecting on seroprevalence in cats (17). In French Guiana, burrowing, granivorous, and insectivorous mammals had much higher prevalences than arboreal mammals (21). Mammals in terrestrial or mixed terrestrial/arboreal habits were more exposed to oocysts than strictly arboreal ones (18, 22).

Among Neotropical primates (NP), the pathology of *T. gondii* varies among infected species. Such contrast in susceptibility to toxoplasmosis could have evolved due to differences in ecology and behavior (23). Toxoplasmosis in the Callitrichinae NP subfamily (*Saguinus, Leontopithecus, Callithrix*) may cause almost 100% mortality, resulting in very low seroprevalence and contributing to difficulty in making ante-mortem diagnosis, particularly in free-ranging populations. The *Saimiri* and *Aotus* genera of Cebidae family and *Ateles* and *Alouatta* in Atelidae family may experience acute and severe toxoplasmosis signs, with mortality from 20 to 80%, allowing for seroprevalence of 15–66% (23). In contrast, signs among *Cebus* sp. NP are usually subacute and moderate, with a very low mortality rate that generates high and persistent immunoglobin G (IgG) titers (24). Reports in captivity range from 28 to 79% of infected *Cebus* sp. monkeys, compared with 30.2% of animals in the wild (24–26). *Cebus* sp. NP may have evolved greater resistance than other NP species after frequent exposure to *T. gondii* (23). *Cebus* sp. NP commonly forage for insects on the ground and drink water from puddles and water holes, where they may encounter oocysts (27). In addition, although most of the *Cebus* diet protein comes from invertebrates, they may consume a variety of vertebrates weighing up to 1/3 of their body weight and constituting up to 3% of their feeding time (27).

In Costa Rica four species of NP have been found. While the Central American white-faced capuchin (*Cebus imitator*) and the Mantled howler monkey (*Alouatta palliata*) are considered at low risk (least concern) according to the International Union for Conservation of Nature (IUCN) Red List, Geoffroy's spider monkey (*Ateles geoffroyi*) and both subspecies of the Central American squirrel monkey (*Saimiri oerstedii oerstedii* and *S. o. citrinellus*) are endangered mainly due to habitat loss and fragmentation (28–32). Some species are still captured for the illegal pet trade, and are, thus, protected from international trade under Appendix I of the Convention on International Trade in Endangered Species (33). However, the population size for these NP species is unknown and has never been estimated. Although Costa Rica has protected areas that cover 25% of the country (34), other conservation actions include a system of incentives to farmers known as payments of environmental services (PES) since 1997 (35), and a national program of biological corridors (PNCB) to increase connectivity between forest patches, species migration and genetic flow (36, 37). *Ex situ* actions include keeping some NP in zoos for education purposes and rescue centers across the country focus on rehabilitation and reintroduction of individuals back to protected areas.

The high mortality rates observed in some NP, which may range from 20 to 80% in *Alouatta* and *Ateles*, can imperil already at-risk populations, but the causes of mortality and high rates of exposure are poorly understood (23, 24), so it is important to assess the infection status in captive NP. Living in altered environments or in contact with humans (where domestic cats are present) can affect NP behavior (38) and exposure to infectious agents (39, 40). Thus, it is important to evaluate impacts of *T. gondii* infection in NP in different areas that experience differential contact with human-altered habitat and have differing behaviors and diet (23, 27). The present study analyzed *T. gondii* exposure in *A. geoffroyi, A. palliata, C. imitator and S. oerstedii* and the influences of environmental, anthropogenic, and biological variables in wild *A. palliata* and *C. imitator* in Costa Rica.

## Materials and Methods

### Ethics

The animal study was reviewed and approved by the Institutional Committee for the Care and Use of Animals (Comité Institucional para el Cuidado y Uso de los Animales) of the Universidad de Costa Rica, and adhered to the legal requirements of Costa Rica. Collection permit number: MINAET-SINAC-Costa Rica: 042-2012-SINAC.

### Study Area

The small tropical country of Costa Rica is located in southern Central America (latitudes 8 02‘26‘ and 11 13‘12‘N, and longitudes 82 33‘48‘ and 85 57‘57‘W) and has an area of 51,100 km^2^ (with around 5% of the biodiversity in the planet). After having one of the world's highest deforestation rates during the 1970s, laws were changed and today, forested areas represent 52% of the country (41), with 25% being protected (34). Precipitation varies seasonally, being the highest from May to early November, and spatially, with wettest regions in the Caribbean slope receiving 3500 to 7000 mm of rainfall per year, and the driest receiving around 900 mm in Guanacaste province, in the Pacific slope (42).

### Sampling

**Figure 1:**
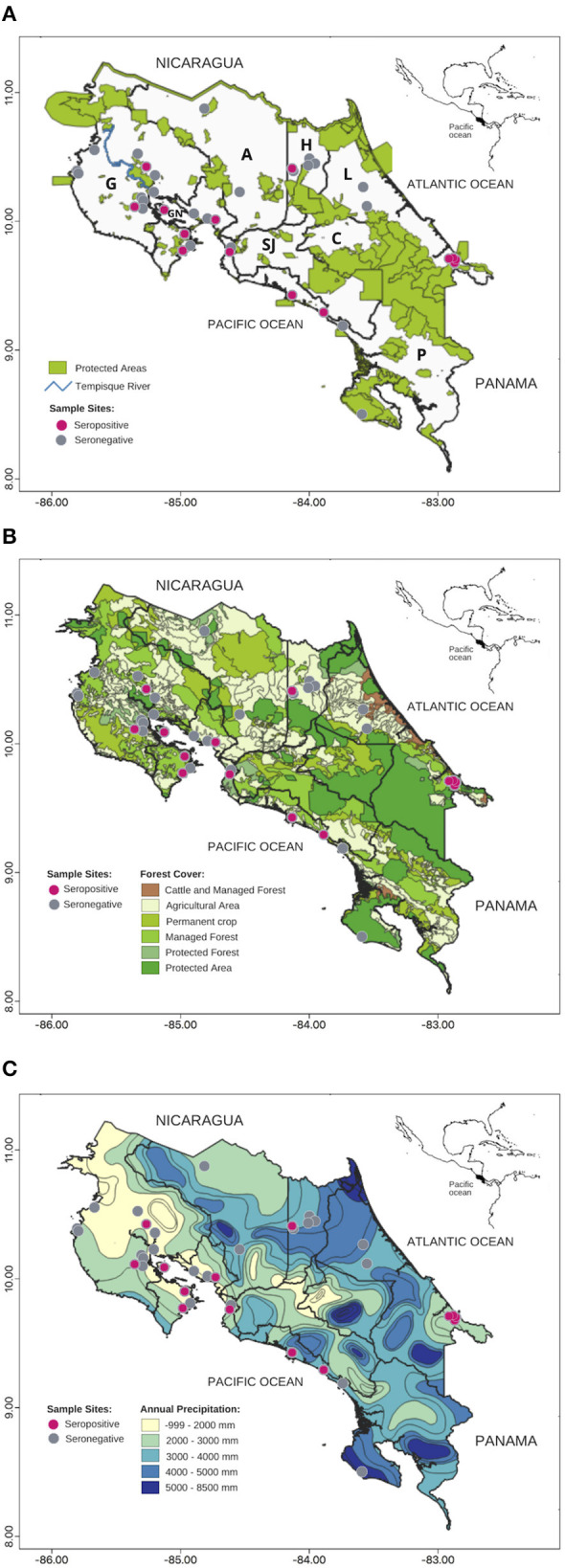
Map of *Toxoplasma gondii* seropositive wild Neotropical primates (NP) from Costa Rica from 2000 to 2015: **(A)** Protected areas, **(B)** Forest Cover, **(C)** Precipitation. Gray circles: seronegative NP, pink circles: seropositive NP. Gulf of Nicoya (GN), Provinces: Alajuela (A), Cartago (C), Guanacaste (G), Heredia (H), Limón (L), San José (SJ), Puntarenas (P).

An observational study was conducted and 245 serum samples were opportunistically collected from four species of native Neotropical primates (NP), *C. imitator, A. palliata, A. geoffroyi*, and *S. oerstedii*, collected throughout Costa Rica in 2000–2015 as part of the project “Epidemiological, genetic, ethological and habitat studies in Costa Rica's monkeys” (Resolution 27-2013- National System of Conservation Areas, Figure 1A). No sample size calculation was previously conducted. Out of the 245 sampled NP, 198 were wild and 47 were in captivity. Most samples from captive animals came from three rescue centers, although 12 different sites were sampled. NP were anesthesized using IM injection of 3.3–11 mg/kg of Zoletil 50^®^ or 5–20 mg/kg ketamine with 0.5–2 mg/kg xylazine (43–45), which for wild NP was loaded into darts (PneuDart. Inc, Type P, 1cc) and delivered using a compressed gas rifle (PneuDart. Inc, model X-Caliber Gauged CO2) targeting the lateral triceps and quadriceps femoris (43). After an individual was anesthetized, blood was sampled from the femoral vein on a plastic tube without anticoagulant, and placed into cooler at 4°C. Once in the laboratory, samples were centrifuged at 2000 RCF for 5 min to separate serum, which was transferred to sterile 1.5 ml tubes at −20°C until processed. Animals underwent a physical examination and were released upon awakening from anesthesia.

### Serological Analysis

A modified agglutination test (MAT) was used to detect IgG specific for *T. gondii* as previously described by Desmonts and Remington (46), and Dubey and Desmonts (47). Formalin-fixed tachyzoites were used as antigen (2 × 10^8^ tachyzoites/mL from KeraFAST Inc., Boston, MA, USA. Catalog No. EH2002). Sera were diluted (1:4) in buffered saline solution (1X PBS) and 50 ul were added to the wells in the first column of 96-well U-bottom microplates. Using a multichannel pipette 25 ul PBS was added to the rest of the wells (columns 2–12) and sera from the first column were serially diluted to 1:8192. Finally, 25 ul of antigen mixture (2.5 ml alkaline buffer, 35 ul 2-mercaptoethanol, 50 ul Evans blue dye (2 mg/ml in H2O) and 150 ul *T. gondii* MAT antigen) were mixed by pipetting and carefully added to each well. The microplates were covered with sealing tape and incubated at 37°C for 24 h and subsequently for 4 h at 4°C. A positive reaction was characterized by a complete “mesh” of agglutination and a clear bottom, while sedimentation of antigen suspension in the form of a blue pellet at the bottom of the well was considered to be a negative reaction. Sera from mice and/or humans were included as positive (titers of 1:200 or greater) and negative controls in each group of processed sera. All antibody titers equal or higher than 1:16 were reported as positive. Titer values from captive vs. wild NP were analyzed using a Mann-Whitney two-tailed test to know if there were significant differences (at a 5% significance level). Pearson's chi-squared test was used to examine differences in individuals within species, sex and serostatus for each group of NP (captive and wild). Small samples (*n* < 6) were assessed with Fisher's exact test. A *p* < 0.05 value was used as significance level, and 95% confidence intervals (CI 95%) were included.

### Biological, Environmental, and Anthropogenic Drivers

Due to insufficient sample size, data for *A. geoffroyi* and *S. oerstedii* were not included for further statistical analyses. Seven independent variables were explored for their association with exposure to *T. gondii* in *A. palliata* and *C. imitator*. Distance to villages, human population size, and human population density in the nearest village were considered indirect indicators of proximal domestic cats. The percentage of forest cover, annual average temperature, and annual precipitation were used as variables that affect oocyst survival. NP sex was used as an intrinsic factor for oocyst exposure (Table 1).

**Table 1:** Independent variables chosen for Generalized Linear Models (GLM) *Toxoplasma gondii* exposure in wild *Alouatta palliata* and *Cebus imitator*.

**Category**	**Variable**	**Units**	**Source**
Intrinsic, Exposure	Sex	Female/male	Field data
Environmental, Oocyst survival	Forest cover percentage (in buffer)	%	Costa Rica Digital Atlas, 2014
	Annual average temperature (buffer)	°C	1 km resolution. Worldclim www.worldclim.org
	Annual precipitation (in buffer)	mm	1 km resolution. Worldclim www.worldclim.org
Anthropogenic, Oocyst dispersion	Distance to towns	m	Costa Rica Digital Atlas, 2014
	Human population (district, year)	Number of people	Population survey. INEC, 2011
	Human population density (district, year)	Number of people/area	Population survey. INEC, 2011

Forest cover, annual average temperature, and annual precipitation were measured within a 1.54 km^2^ circular area (700 m radius) that was constructed around each sample's geolocation point (QGIS software, version 2.14). Data for these variables were obtained from Costa Rica Digital Atlas (2014) and WorldClim (48). The buffer size aimed to include both species' home ranges and adjacent areas (49).

Generalized linear models (GLM) with binomial distribution and logit link were run in R V3.4.2 (50) to explore associations between predictor variables and *T. gondii* seropositivity. The original predictors (forest cover and precipitation) were centered by subtracting their means and scaled by dividing by their standard deviations. The most parsimonious model was chosen by selecting the one with the lowest Akaike information criterion (AIC). Akaike weights (wi) were calculated to determine the evidence in favor of each model and estimate the relative importance of variables (Table 2).

**Table 2:** Generalized Linear Models that explain intrinsic, environmental, and anthropogenic attributes on *Toxoplasma gondii* seroprevalence in *Alouatta palliata* and *Cebus imitator*.

**Model**	**Variables**	K*	**AIC**	**ΔAIC**	**w_**i**_**
***A. palliata***					
1	Forest cover + precipitation	3	44.784	0	0.242
2	Precipitation	2	44.827	0.04	0.237
3	Population density + precipitation	3	46.103	1.32	0.125
4	Population density + forest cover + precipitation	4	46.241	1.46	0.117
5	Sex + precipitation	4	46.683	1.9	0.094
6	Sex + forest cover + precipitation	5	46.784	2	0.089
7	Sex + population density + precipitation	5	47.885	3.1	0.051
8	Sex + population density + forest cover + precipitation	6	48.238	3.45	0.043
9	Sex	3	73.590	28.8	1.35E-07
10	Sex + forest cover	4	73.770	29	1.23E-07
11	Sex + population density	4	74.368	29.6	9.13E-08
12	Forest cover	2	75.220	30.4	5.97E-08
13	Sex + population density + forest cover	5	75.235	30.45	5.92E-08
14	Null	1	75.616	30.8	4.89E-08
15	Population density	2	76.804	32	2.70E-08
16	Population density + forest cover	3	76.958	32.2	2.50E-08
***C. imitator***					
1	Precipitation	2	39.724	0	0.303
2	Forest cover + precipitation	3	40.815	1.09	0.176
3	Population density + precipitation	3	41.223	1.5	0.143
4	Sex + precipitation	4	41.457	1.7	0.127
5	Sex + forest cover + precipitation	5	42.524	2.8	0.075
6	Population density + forest cover + precipitation	4	42.774	3.05	0.066
7	Sex + population density + precipitation	5	42.993	3.3	0.059
8	Sex + population density + forest cover + precipitation	6	44.449	4.7	0.028
9	Null	1	47.033	7.3	7.8E-03
10	Forest cover	2	48.636	8.9	3.5E-03
11	Population density	2	49.026	9.3	2.9E-03
12	Sex	3	49.032	9.3	2.9E-03
13	Population density + forest cover	3	50.490	10.77	1.4E-03
14	Sex + forest cover	4	50.636	10.9	1.3E-03
15	Sex + population density	4	51.025	11.3	1.07E-03
16	Sex + population density + forest cover	5	52.489	12.8	5.1E-04

* *K = Number of parameters per model, including the intercept*.

Each NP species was modeled separately. Multicollinearity among the seven variables was evaluated by means of the variance inflation factor (VIF). When analyzing all the proposed variables, a very high VIF value (>10) was obtained suggesting multicollinearity, therefore the model was reduced to four variables (sex, forest cover, annual precipitation, and human population density). With these models, none of the variables obtained a VIF value <10 (Table 2).

## Results

*Toxoplasma gondii*-specific IgG antibodies were found in 59.6% (28/47, 95% CI = 44.27–73.63) of captive NP and 11.6% (23/198, 95% CI = 7.7–17.34) of wild NP. In captive NP, seroprevalence was high for both species: 59.1% (26/44, 95% CI = 43.25–73.66) of *A. geoffroyi* and 66.7% (2/3, 95% CI = 9.43–99.16) of *C. imitator*. There were no significant differences in species or sex with 58.6% (17/29, 95% CI = 38.94–76.47) of *A. geoffroyi* females and 60.0% (9/15, 95% CI = 32.29–83.66) males being positive, while for *C. imitator*, 50.0% (1/2, 95% CI = 1.26–98.74) females and 100% (1/1, 95% CI = 2.5–100) male presented positive results (Tables 3, 4).

**Table 3:** Prevalence of *Toxoplasma gondii* antibodies in captive and wild Neotropical primates in Costa Rica according to species and sex.

**Captivity positives (IC 95%)**			
**Species**	**Female** **n-%**	**Male** **n-%**	**Total** **n-%**
*A. geoffroyi*	17/29–58.6%	9/15–60%	26/44–59.1%
	(IC = 38.94–76.47)	(IC = 32.29–83.66)	(IC = 43.25–73.66)
*C. imitator*	1/2–50%	1/1–100%	2/3–67%
	(IC = 1.26–98.74)	(IC = 2.5–100)	(IC = 9.43–99.16)
**Total**	18/31–58.06%	10/16–62.5%	28/47–59.6%
	(IC = 39.07–75.45)	(IC = 35.43–84.8)	(IC = 44.27–73.63)
**Wild positives (IC 95%)**			
**Species**	**Female**	**Male**	**Total**
	**n-%**	**n-%**	**n-%**
*A. palliata*	8/76–10.53%	2/75–2.66%	10/151–6.62%
	(IC = 4.65–19.69)	(IC = 0.32–9.3)	(IC = 3.22–11.84)
*A. geoffroyi*	2/3–66.67%	0/2–0%	2/5–40%
	(IC = 9.43–99.16)	(IC = 0–84.19)	(IC = 5.27–85.34)
*C. imitator*	3/10–30%	8/27–29.63%	11/37–29.73%
	(IC = 6.67–65.24)	(IC = 13.75–50.18)	(IC = 15.88–46.98)
Total	13/89–14.6%	0/104–9.61%	193–11.92%
	(IC = 8.01–23.68)	(IC = 4.7–16.97)	(IC = 7.7–17.34)

*Samples of Saimiri oerstedii (n = 5) were excluded*.

**Table 4:** Seroprevalence of *Toxoplasma gondii* in captive Neotropical Primates from Costa Rica according to site and species.

**Province**	**Site**	**Species**	**Positives/sample**	**Seroprevalence %**
Alajuela	Cap 1	*A. geoffroyi*	13/19	68.40
	Cap 2	*A. geoffroyi*	0/1	0
	Cap 3	*A. geoffroyi*	1/2	50
	Cap 4	*A. geoffroyi*	3/3	100
Guanacaste	Cap 5	*A. geoffroyi*	0/2	0
Heredia	Cap 6	*C. imitator*	1/2	50
	Cap 7	*C. imitator*	1/1	100
Limon	Cap 8	*A. geoffroyi*	1/1	100
	Cap 9	*A. geoffroyi*	1/2	50
Puntarenas	Cap 10	*A. geoffroyi*	3/7	42.80
	Cap 11	*A. geoffroyi*	4/6	66.70
San Jose	Cap 12	*A. geoffroyi*	0/1	0
**Total**		*A. geoffroyi*	26/44	59.10
		*C. imitator*	2/3	66.70
		**Total**	**28/47**	**59.60**

In contrast, there was a significant difference in seroprevalence among wild NP species (*X*^2^ = 20.072; df = 3; *p* = 0.000164): *A. geoffroyi* had the highest seroprevalence with 40% (2/5, 95% CI = 5.27–85.34), followed by *C. imitator* with 30% (11/37, 95% CI = 15.88–46.98) and *A. palliata* with 6.6% (10/151, 95% CI = 3.22–11.84), while no *S. oerstedii* were positive (0/5). No significant difference was found regarding sex for any of the species in the wild group; however, greater seroprevalence was observed for females in the three positive species (Tables 3, 5).

**Table 5:** Seroprevalence of *Toxoplasma gondii* in wild Neotropical Primates from Costa Rica according to sample site and species.

**Province**	**Canton**	**District**	**Species**	**Positives/Sample**	**Seroprevalence %**
Alajuela	Los Chiles	Cano Negro	*A. palliata*	0/1	0
	San Ramon	Angeles	*A. palliata*	0/4	0
Guanacaste	Bagaces	Bagaces	*A. palliata*	1/19	5.26
	Bagaces	Bagaces	*C. imitator*	0/1	0
	Canas	Bebedero	*A. palliata*	0/2	0
	Carrillo	Sardinal	*A. palliata*	0/3	0
	Nicoya	Mansion	*A. palliata*	1/11	9
	Nicoya	Quebrada Honda	*A. palliata*	0/8	0
	Santa Cruz	Tempate	*A. palliata*	0/7	0
Heredia	Sarapiqui	Horquetas	*C. imitator*	0/2	0
	Sarapiqui	La Virgen	*A. palliata*	0/1	0
	Sarapiqui	La Virgen	*A. geoffroyi*	2/2	100
	Sarapiqui	La Virgen	*C. imitator*	0/3	0
	Sarapiqui	Puerto Viejo	*A. palliata*	0/11	0
	Sarapiqui	Puerto Viejo	*C. imitator*	0/1	0
Limon	Guacimo	Rio Jimenez	*A. geoffroyi*	0/1	0
	Siquirres	Cairo	*A. palliata*	0/5	0
	Talamanca	Cahuita	*A. palliata*	4/28	14
Puntarenas	Chomes	Chomes	*A. palliata*	0/9	0
	Garabito	Tarcoles	*A. palliata*	0/1	0
	Garabito	Tarcoles	*C. imitator*	2/2	100
	Golfito	Puerto Jimenez	*A. palliata*	0/3	0
	Golfito	Puerto Jimenez	*C. imitator*	0/2	0
	Golfito	Puerto Jimenez	*S. oerstedii*	0/5	0
	Osa	Bahia Ballena	*A. palliata*	0/6	0
	Puntarenas	Barranca	*C. imitator*	1/4	25
	Puntarenas	Cobano (IslaN)	*A. geoffroyi*	0/2	0
	Puntarenas	Cobano (Curu)	*C. imitator*	4/11	36.40
	Puntarenas	Isla Chira	*A. palliata*	1/2	50
	Puntarenas	Pitahaya	*A. palliata*	0/4	0
	Puntarenas	San Lucas	*A. palliata*	3/23	13
	Quepos	Quepos	*A. palliata*	0/3	0
	Quepos	Quepos	*C. imitator*	3/7	42.86
	Quepos	Savegre	*C. imitator*	1/4	25
**Total**			*A. palliata*	9/151	6
			*A. geoffroyi*	2/5	40
			*C. imitator*	11/37	29.70
			*S. oerstedii*	0/5	0
			**Total**	**23/198**	**11.60**

**Figure 2:**
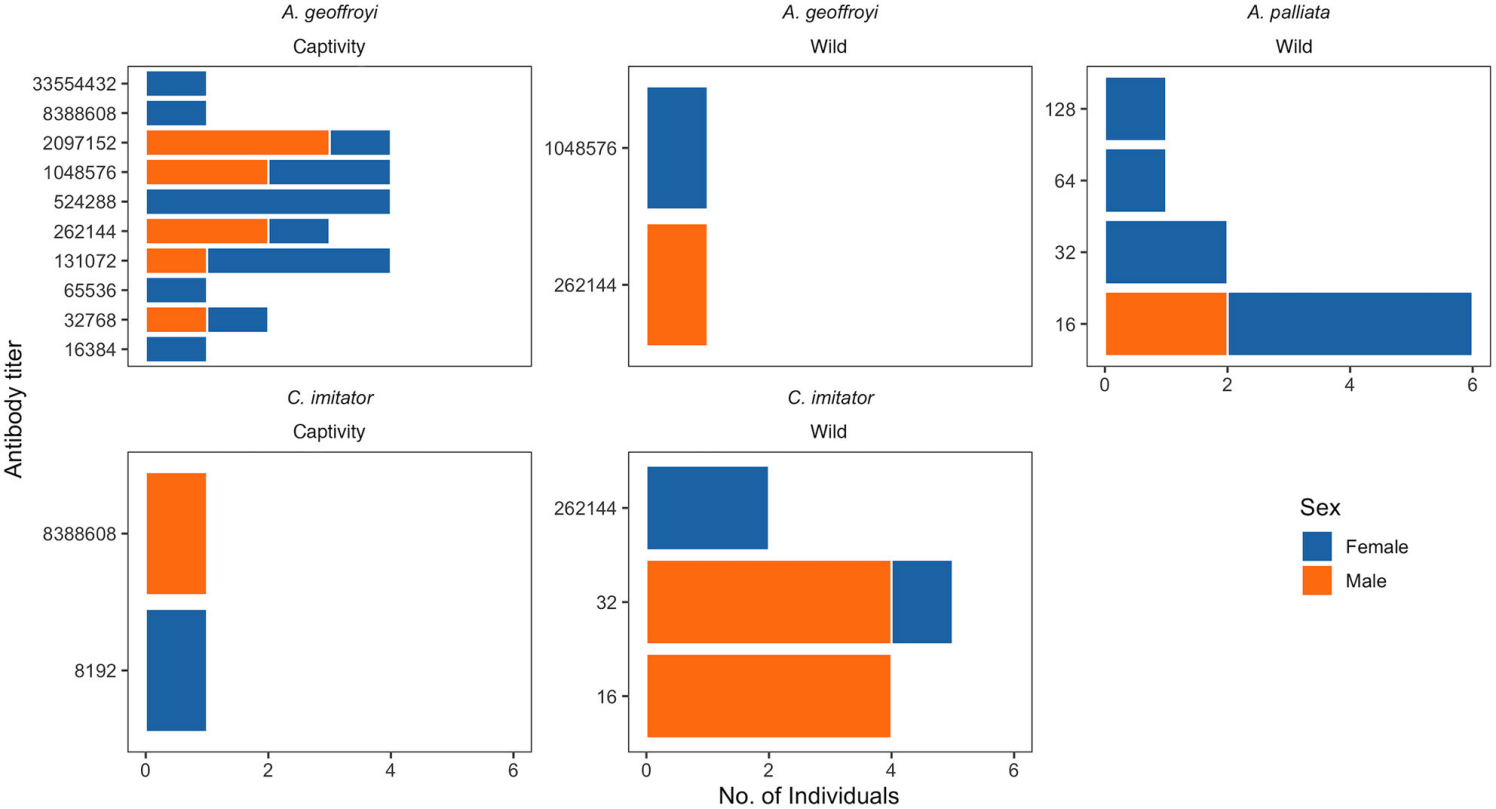
Antibody titers of *Toxoplasma gondii* IgG in Neotropical primates of Costa Rica.

Antibody titers tended to be relatively low (Median = 8) in wild NP (<1:128), with the exception of four individuals: one *A. geoffroyi* and two *C. imitator* with antibody titers of 1:262,144 and one *A. geoffroyi* with a titer of 1:1,048,576). In contrast, antibody titers were considerably higher (Median = 262,144) in captive NP, from 1:8,192 to 1:33,554,000 (Figure 2). These two groups of NP were statistically different in their titer values (*p* < 0.00001).

The model that best accounted for seropositivity among *A. palliata* (minimizing AIC) was forest cover and annual precipitation (Figure 1B and Table 2), with a positive relationship between forest cover and seropositivity and inverse relationship with annual precipitation. However, evidence weights (wi) for the second and third best-supported models were 1.02 and 1.94 times lower than model 1 respectively, which suggests that no one model is strongly supported as the best candidate. The second best model was annual precipitation (ΔAIC = 0.04) and the third was for human population density and annual precipitation (ΔAIC = 1.32). Notably, the precipitation parameter appeared among the top eight models.

For *C. imitator*, annual precipitation alone best accounted for seropositivity with a significant inverse relationship (Figures 1C, 2 and Table 2). The next best models were forest cover and annual precipitation (ΔAIC = 1.09) and human population density and precipitation (ΔAIC = 1.5), with evidence weights 1.73 and 2.12 times lower than model 1, respectively. Again, the effect of precipitation on seropositivity was important for *C. imitator* when comparing the sum of the weights of Akaike (wi) in the models that contained this variable (0.98) with respect to the others, such as forest cover (0.35), population density (0.3), and sex (0.29).

## Discussion

The present survey documented widespread exposure and species-specific risks of *T. gondii* infection. The seroprevalence obtained for captive NP is similar to other studies in South America, such as 30.8 (26) and 76% (51) using MAT in captive *Cebus apella*, and 79% in *Cebus* sp. and 57% in *Ateles* sp. from zoos in São Paulo, Brazil (24). The high prevalence of NP in captivity may be due to management practices, including improperly washed fruits or vegetables, and raw or undercooked meat (51, 52), proximity of wild or domestic cats, and invasion of enclosures by infected birds and rodents that might be ingested by NP (51, 52). These sources of infection would be similar for male and female NP in captivity, consistent with our findings.

The 11.6% seroprevalence in wild Costa Rican NP was lower than the 26.6% (16/60) reported by Garcia et al. (25) using MAT in Brazil, and the prevalence we found for *A. palliata* (6.6%) is lower than documented for *Alouatta caraya* by Garcia et al. (25) in Paraná (17%, 3/17) and Molina et al. (53) in São Paulo, Brazil (75%, 15/20). However, it is higher than findings by de Thoisy et al. (54), who found only 4% (2/50) seropositive *Alouatta senilicus* in French Guiana, but similar in the observed proportion of positive females. The present data did not indicate a significant difference between sexes for any of the species, although this should be studied further given the sex bias observed in French Guiana and among domestic cats (17).

In the case of *C. imitator*, the prevalence is similar to that reported by Garcia et al. (25) in *Cebus* spp. with 30.2% (13/43). In fact, several studies have identified that carnivorous diet is a risk factor for *T. gondii* infection (55, 56). On the other hand, the prevalence found in *A. geoffroyi* (40%, 2/5) appears to be high, which is surprising considering its arboreal and herbivorous (frugivorous) behavior, characteristics that minimize its exposure to the parasite. No prevalence reports were found for wild *Ateles* sp. to compare this finding to. However, sample sizes for both *A. geoffroyi* and *S. oerstedii* were small, so these findings should be interpreted with caution. The difference in seropositivity observed between howler (*A. palliata*) and capuchin monkeys (*C. imitator*) coincides with Garcia et al. (25) and corresponds to behavioral characteristics between species. Howlers are predominantly folivorous, supplementing their diet with fruits, flowers and seeds, and obtaining most of the water they need from their food [reviewed by (49)]. However, they can drink water accumulated in branches, trunks or bromeliads (57), or search for it on the ground (58). The source of infection for howlers would be water bodies infected with oocysts. Meanwhile, capuchins are the most omnivorous NP, feeding on various sources like fruits, insects and small vertebrates such as birds, rodents, squirrels, coatis, bats, frogs, and lizards [reviewed by (23, 49)]. They frequently go down to the undergrowth and ground while foraging and traveling (59). In addition, *Cebus* spp. drink water directly from puddles (27). These characteristics give capuchins greater exposure to oocysts in the soil, water, in invertebrates (transport hosts), and to tissue cysts present in infected vertebrates.

The low antibody titers obtained for the wild NP (1:16 and 1:32) coincide with those reported by Garcia et al. (25), who found mostly 1:16 and 1:32 for a single individual. While *A. palliata* had low antibody titers, two *A. geoffroyi* and two *C. imitator* had very high antibody titers (1:262,144–1:1,048,576). Molina et al. (53) reported values of 1:25 for *Callithrix penicillata* (a very susceptible species) and higher titers (up to 1:1,600) for *A. caraya*, arguing that differences in prevalence and titers could respond to differences in host susceptibility, contact rates or post-exposure time. Indeed, *Alouatta*'s susceptibility in comparison to *Cebus* could be reflected in serological differences, since the probability of post-infection survival and, therefore, developing an immune response is naturally lower for howlers. Furthermore, if these individuals die, they would be excluded from the population and the sample, reducing the number of individuals captured with high titers. In contrast, titers of captive NP were high in this study. Leite et al. (26) reported antibody titers of 1:8,000 by MAT for *Cebus sp*. in captivity, while Ekanayake et al. (40) reported antibody titers >1:256 (up to 1:4,096) in 3 of 21 positive free-ranging but urban macaques in Sri Lanka. Management factors along with the increased life expectancy for *Cebus* sp. and *Ateles* sp. in captivity could explain the chance of infection and high titers observed in this group.

It is worth highlighting the large number of positive samples from the Gulf of Nicoya (Figure 1A). Tempisque River is one of the most important basins in the country, draining 10.6% of the territory and flowing into the Gulf of Nicoya (60). Areas close to bodies of water could represent sources of infection for NP, because water can be contaminated at any point, transport oocysts long distances, and favor their survival (2). Samples from protected areas with high levels of human contact also showed high prevalence and high titers in the two wild *C. imitator* mentioned before. In certain national parks and private reserves, feeding wild monkeys is a common practice, and capuchin and squirrel monkeys sometimes exhibit agonistic behaviors that include taking food directly from humans and coming down to the ground. Such opportunistic behavior (61) may increase exposure to infectious agents (39, 40).

Besides identifying specific areas where there could be elevated risk of infection, this data indicates that environmental variables such as forest cover and precipitation could be associated with exposure risk. Seropositivity in *A. palliata* was higher when there was a higher percentage of forest cover and less annual precipitation. Forest cover can protect oocysts from sunlight, allowing them to remain viable for 1–1.5 years, when protected (62, 63). Smith and Frenkel (64) and Almería et al. (12) found greater seroprevalence in hares and other mammals sampled in forested areas vs. more arid grasslands, arguing that shadow and relative humidity provided by forest cover act on oocyst conservation by decreasing the evaporation rate and desiccation of oocysts.

Wet seasons tend to increase oocyst survival (63). In fact, *T. gondii* seroprevalence in cats (15) and humans (11) has been associated with rainy and warm episodes (North Atlantic Oscillation), and in wild ruminants (13) with humid areas. Contrary to expectations, in this study precipitation was inversely related with seropositivity in both NP species. Costa Rica is a tropical country with high relative humidity and stable temperature overall. It is possible that increased precipitation in Costa Rica results in greater runoff, transporting oocysts toward the coasts and away from the animals. In recent past years, low rainfall due to the El Niño phenomenon has generated severe droughts and forest fires in some areas of Costa Rica. Among many other animals, *A. palliata* were severely affected by water and food shortages, with high mortality due to dehydration and starvation, as well as injuries due to troops fighting for food. Because behavior change driven by droughts can increase exposure to parasites present in the scarce sources of water, the risk of disease increases especially for animals weakened by starvation and dehydration.

In the present study, the source of exposure of wild NP could be wild and not domestic cats, which might explain the low effect of human population density on seropositivity. Contact with humans has been associated with high seropositivity in macaques (40), which can become infected by ingesting human food from the ground in areas frequented by domestic cats (3). Domestic cat population estimates as well as sampling in areas where humans feed and interact with wildlife should be included in future studies.

Additionally, *Toxoplasma* genotypes produce different degrees of virulence in humans and mice (65), and given the high diversity recently found in Central and South America (Ajzenberg et al., 2004; Lehmann et al., 2004) (66–69) the observed differences in seroprevalence between wild and captive NP might be due to different genotypes. Little is known about the genotypes circulating in wildlife, and associations between strain type, lesion patterns, and clinical outcome have not been reported in wildlife frequently. Thus, future studies that focus on genotyping and virulence of *T. gondii* isolates in wildlife and domestic animals from wild and anthropized environments would be of great value.

Antibody titer of 1:25 is often considered as evidence of exposure to *T. gondii* in many mammals (70) and 1:5 in birds (71). However, there is no antibody titer that is considered specific for primates to maximize sensitivity of detection given the high susceptibility and low seroprevalence in some NP species, we reported all antibody titers of 1:16 or higher (25).

**Figure 3:**
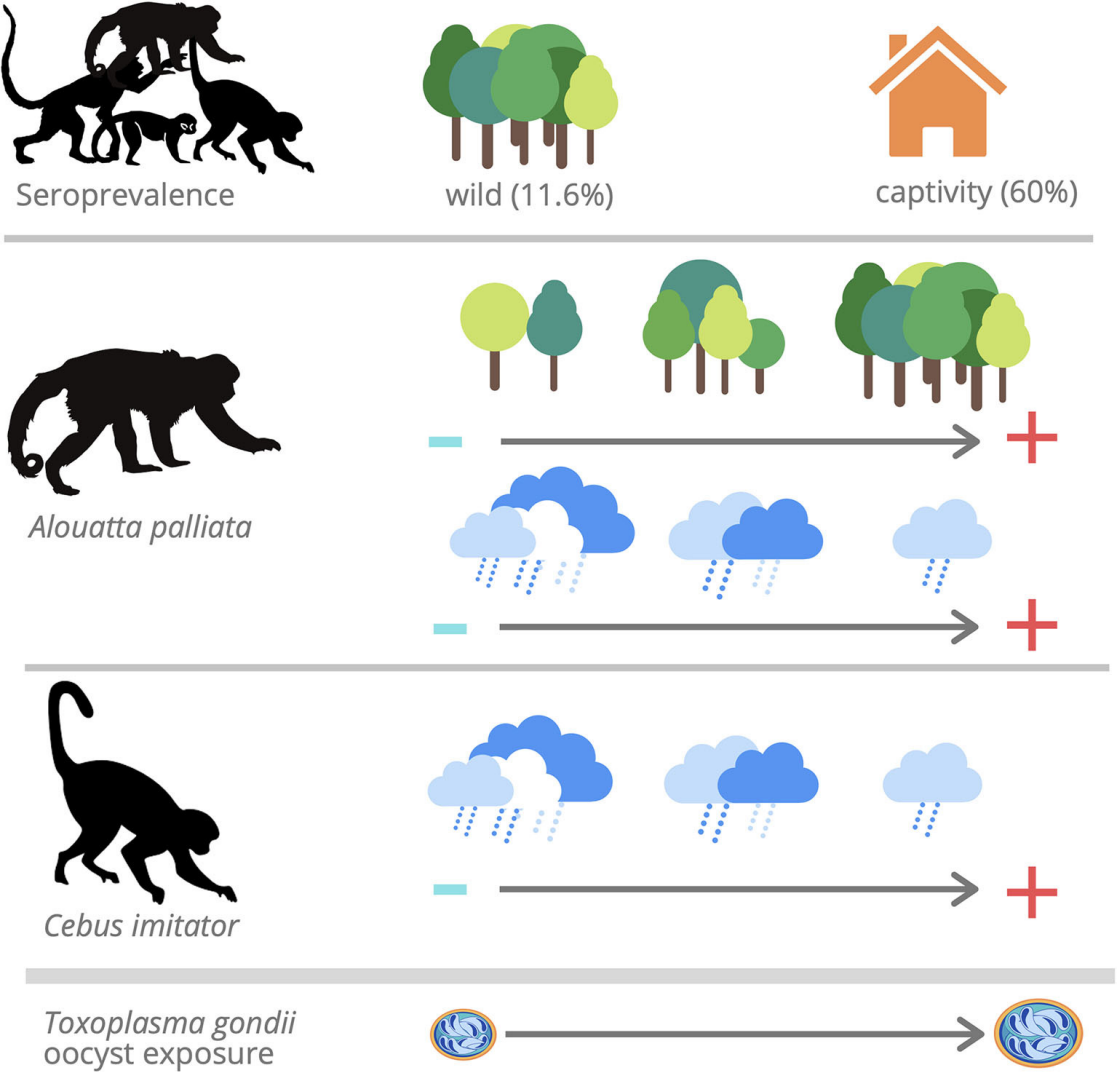
*Toxoplasma gondii* IgG antibodies were found in 59.6% (28/47) of captive NP and 11.6% (23/198) of wild NP. The model that best accounted for seropositivity among *Aloutta palliata* with a positive relationship between forest cover and seropositivity and inverse relationship with annual precipitation. The model that best accounted for seropositivity among *Cebus imitator* with an inverse relationship with annual precipitation.

This study documented widespread *T. gondii* infection in NP and species-specific risks of infection for the first time in Costa Rica (Figure 3). The high seroprevalence and titers found in captive capuchin and spider monkeys may be due to management practices, the proximity of cats or intermediate hosts, and the increased life expectancy in captivity for these species. Because the number of samples was small, especially in the case of *C. imitator*, further studies should assess these findings. The low seroprevalence and titers in wild NP varied between species. The difference in seropositivity observed between wild howler (*A. palliata*) and capuchin monkeys (*C. imitator*) in this study agrees with behavioral and dietary characteristics, in which capuchins are more exposed to oocysts while foraging on the ground, and by ingesting invertebrates (transport hosts) and vertebrates (tissue cysts). However, *Alouatta*'s susceptibility compared to *Cebus* might also explain the observed serological differences, due to decreased survival. These data indicated that specific areas could represent an elevated risk of infection (i.e., water runoff and human interaction), and environmental variables such as abundant forest cover and low precipitation could be associated with higher exposure risk in wild NP. Surveillance of *T. gondii* in NP is required to better understand the infection status, genotypes, and drivers involved in wild and captive NP, including individuals in the process of reintroduction, so that biosecurity measures are improved, avoiding the release of infected individuals, and developing novel strategies to protect wild populations.

## Data Availability Statement

The raw data supporting the conclusions of this article will be made available by the authors, without undue reservation.

## Ethics Statement

The animal study was reviewed and approved by the Institutional Committee for the Care and Use of Animals (Comité Institucional para el Cuidado y Uso de los Animales) of the Universidad de Costa Rica, and adhered to the legal requirements of Costa Rica. Collection permit number: MINAET116 SINAC-Costa Rica: 042-2012-SINAC.

## Author's Note

*Toxoplasma gondii* is a widespread zoonotic protozoan that has a wide host range, which includes birds and mammals. It uses different routes and modes of transmission to move from their definitive hosts (domestic and wild felids) to the intermediate hosts. Even if the *T. gondii* infections usually produce low or any symptoms, in Neotropical primates it is a severe disease with a high rate of mortality. However, there are scarce studies for these species of monkeys, and the factors associated their infection in wildlife. The oocysts are the infective stage for intermediate hosts; they are excreted by felids in their feces, and maintained in the environment waiting for an intermediate host. For that reason it is important to determine the influences of environmental, anthropogenic, and biological variables for the exposure of *T. gondii* in Neotropical primates. In our study we found that forest cover is associated with the presence of *Alouatta palliata* seropositives, and inversely related to annual rainfall with Alouatta palliata and Cebus imitator seropositives. The difference in exposure between monkey species reflects influence of ecology and behavior. High antibody titers in captivity suggest increased exposure due to management.

## Author Contributions

CN and AC conceived the investigation and wrote the article. AC and GG-E collected samples. CN, NR, and CS conducted the laboratory analysis. CN, MS, CI-C, AC, and OR-C performed the statistical analysis. JF and GS copy reviewed and edited. All authors reviewed the manuscript.

## Conflict of Interest

The authors declare that the research was conducted in the absence of any commercial or financial relationships that could be construed as a potential conflict of interest.
